# Regenerative Injections Including 5% Dextrose and Platelet-Rich Plasma for the Treatment of Carpal Tunnel Syndrome: A Systematic Review and Network Meta-Analysis

**DOI:** 10.3390/ph13030049

**Published:** 2020-03-18

**Authors:** Chih-Peng Lin, Ke-Vin Chang, Yi-Kai Huang, Wei-Ting Wu, Levent Özçakar

**Affiliations:** 1 Department of Anesthesiology, National Taiwan University Hospital and National Taiwan University College of Medicine, Taipei 10048, Taiwan; cplin0123@gmail.com; 2 Department of Physical Medicine and Rehabilitation, National Taiwan University Hospital and National Taiwan University College of Medicine, Taipei 10048, Taiwan; 3 Department of Physical Medicine and Rehabilitation, National Taiwan University Hospital, Bei-Hu Branch, Taipei 10845, Taiwan; wwtaustin@yahoo.com.tw; 4 Department of Physical Medicine and Rehabilitation, National Taiwan University Hospital, Hsin-Chu Branch, Hsin-Chu 30059, Taiwan; icq164@gmail.com; 5 Department of Physical and Rehabilitation Medicine, Hacettepe University Medical School, Ankara 06100, Turkey; lozcakar@yahoo.com

**Keywords:** median nerve, entrapment, regeneration, platelet-rich plasma, dextrose, steroid

## Abstract

This network meta-analysis aimed to integrate the available direct and indirect evidence on regenerative injections—including 5% dextrose (D5W) and platelet-rich plasma (PRP)—for the treatment of carpal tunnel syndrome (CTS). Literature reports comparing D5W and PRP injections with non-surgical managements of CTS were systematically reviewed. The main outcome was the standardized mean difference (SMD) of the symptom severity and functional status scales of the Boston Carpal Tunnel Syndrome Questionnaire at three months after injections. Ranking probabilities of the SMD of each treatment were acquired by using simulation. Ten studies with 497 patients and comparing five treatments (D5W, PRP, splinting, corticosteroid, and normal saline) were included. The results of the simulation of rank probabilities showed that D5W injection was likely to be the best treatment, followed by PRP injection, in terms of clinical effectiveness in providing symptom relief. With respect to functional improvement, splinting ranked higher than PRP and D5W injections. Lastly, corticosteroid and saline injections were consistently ranked fourth and fifth in terms of therapeutic effects on symptom severity and functional status. D5W and PRP injections are more effective than splinting and corticosteroid or saline injection for relieving the symptoms of CTS. Compared with splinting, D5W and PRP injections do not provide better functional recovery. More studies investigating the long-term effectiveness of regenerative injections in CTS are needed in the future.

## 1. Introduction

As the most common peripheral entrapment neuropathy, carpal tunnel syndrome (CTS) affects approximately 3.8% of the general population [1], with an incidence of around 300 cases per 100,000 person-years [2]. CTS leads to clinical symptoms including pain, numbness, tingling sensation, and weakness in the hand innervated by the median nerve. A recent observational study showed that painful symptoms were more likely to develop near the carpal tunnel, whereas non-painful sensory disturbances were identified to distribute more peripherally in patients with CTS [3]. The risk factors of CTS include, but are not limited to, female sex, obesity, diabetes, and hypothyroidism [4,5]. The diagnosis of CTS is traditionally based on electrophysiological tests; however, ultrasonographic nerve cross-sectional area measurements have also recently emerged as useful alternatives [6]. The therapeutic algorism for CTS can be based on symptom severity, findings of electrophysiological tests, and responsiveness to non-surgical therapy. Conservative treatments for mild to moderate CTS include corticosteroid injection [7], splinting [8], and physiotherapy (like therapeutic ultrasound, exercise, and neural mobilization) [9,10]. In patients with moderate to severe symptoms and poor responsiveness to non-operative management, carpal tunnel release (CTR) is indicated [11]. However, the symptoms might persist in 3% to 20% of patients after CTR [12]. In recalcitrant cases, the physicians should consider surgical management, such as revision neuroplasty, neurolysis, and nerve reconstruction [12].

Corticosteroids function as potent anti-inflammatory medications and have been widely used as the main injectate for treating CTS. A recent randomized controlled trial that recruited 234 patients with CTS demonstrated that a single corticosteroid injection was better than night splinting in terms of clinical effectiveness at six weeks post-injection [13]. The aforementioned result is consistent with that of a previous systematic review that revealed the short-term (at one month) benefits of corticosteroid injection in the treatment of CTS [14]. Injected corticosteroid is known to have an effective duration of 1–4 weeks in the target tissue [15]; however, its long-term advantage for CTS is also not supported by the available literature [14].

Regenerative medicine techniques, which involve regenerating human cells, tissues, or organs to restore normal function, have been increasingly used in the treatment of various musculoskeletal disorders [16]. In this regard, dextrose and platelet-rich plasma (PRP) are the two most commonly used regenerative injection regimens, and numerous in vitro and in vivo studies have shown their potential role in promoting tissue repair [17,18]. Furthermore, the pathophysiology of CTS comprises increased intra-compartment pressure and microcirculatory disturbance in subsynovial connective tissue [19]. An animal study revealed that chronic nerve compression could lead to a decrease in intermodal length and myelin thickness of Schwann cells [20]. As certain neurodegenerative pathologies, such as demyelination and axonal degeneration, have been observed in the median nerves of patients with CTS [21], perineural regenerative injection may have better and longer effectiveness than traditional non-surgical treatments. To this end, although several randomized controlled trials have shown the promising effects of 5% dextrose (D5W) and PRP injections on CTS [22,23], the reported clinical outcome is inconsistent across different studies. Therefore, the present systematic review and network meta-analysis aimed to synthesize and compare the available direct and indirect evidence on regenerative injections, including D5W and PRP, for treating CTS.

## 2. Results

### 2.1. Study Selection and Characteristics of the Included Studies

**Figure 1:**
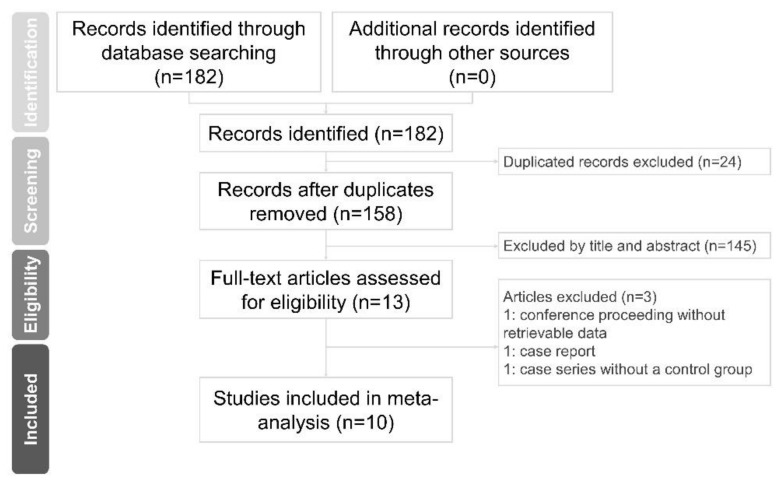
Flow diagram showing the study selection process based on the suggested format in the PRISMA (Preferred Reporting Items for Systematic Reviews and Meta-Analyses) guidelines.

A total of 182 records were found in the initial literature search. We retrieved 13 articles for a full-text review, after eliminating duplicates and irrelevant citations. Three studies were further discarded because one was a conference proceeding without retrievable data [24], one was a case report [25], and the other was a case series without a control group [26]. Therefore, ten studies were included in the final quantitative analysis (Figure 1) [22,23,27,28,29,30,31,32,33,34].

We summarized the characteristics of the included studies in Table 1 and Table 2. This meta-analysis included 497 patients with 518 affected wrists. The majority (> 75%) of the overall participants were women. The average age in the patient group across the included trials ranged from 36.6 to 60.4 years. The average symptom duration ranged from 13.7 to 72.0 months.

Of the ten included studies, three compared PRP with corticosteroid injection [27,30,32], one compared PRP with saline injection [28], three compared PRP injection (with or without additional splinting) with splinting alone [23,29,33], one [34] compared D5W with saline injection, one [22] compared D5W with corticosteroid injection, and one [31] compared PRP with D5W injection. With respect to outcome assessment, the Boston Carpal Tunnel Questionnaire (BCTS) was used in nine [22,23,27,29,30,31,32,33,34] of the ten enrolled studies. The visual analogue scale (VAS) for pain was used as a surrogate for the symptom severity scale (SSS) in one trial [28]. In terms of the injection technique, seven studies [22,23,28,30,31,33,34] used ultrasound guidance, whereas three [27,29,32] employed palpation guidance. The maximal follow-up duration was 24 weeks in five trials [22,31,32,33,34], 12 weeks in three trials [27,28,30], 10 weeks in one trial [29], and four weeks in one trial [23]. Only one study [29] reported some minor adverse effects after the injection.

### 2.2. Assessment of the Quality of the Included Studies

**Figure 2:**
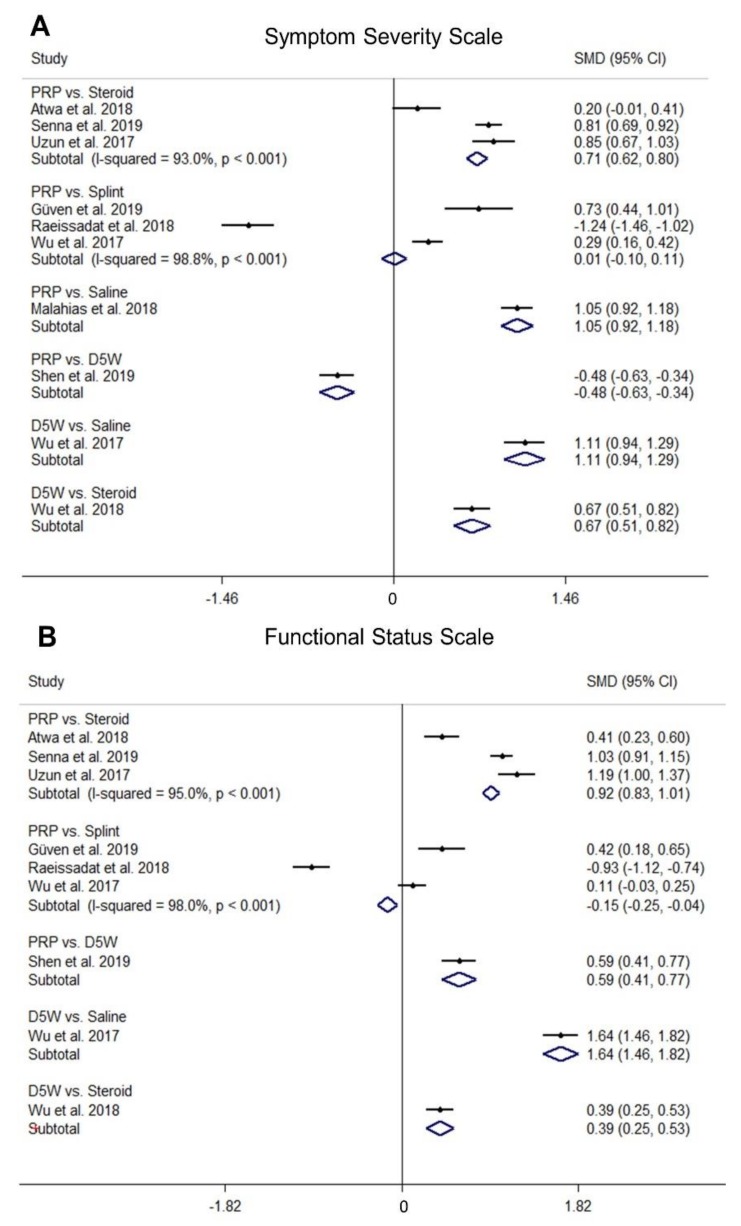
Forest plot of pairwise comparisons of the standardized mean difference (SMD) between different subgroups in terms of the (**A**) symptom severity scale and (**B**) functional status scale of the Boston Carpal Tunnel Questionnaire. *PRP, platelet-rich plasma; D5W, 5% dextrose.*

We summarized the quality assessment in Figure 1 and Figure 2. In most of the included studies, the item found to have a high risk of bias was blinding of participants and personnel. The aforementioned procedure was not applicable in studies in which splinting was used in the comparative arms. Likewise, for the PRP trials, the patients were aware of which treatment they received if no sham blood withdrawal was used in the control injection groups.

On the other hand, selective reporting was revealed to have a low risk of bias in the majority of the enrolled trials. Almost all of the studies reported the outcome by using acceptable clinical assessment tools, together with electrophysiological examinations.

### 2.3. Comparison of Standardized Mean Differences (SMDs) of SSS among Different Treatments

**Figure 3:**
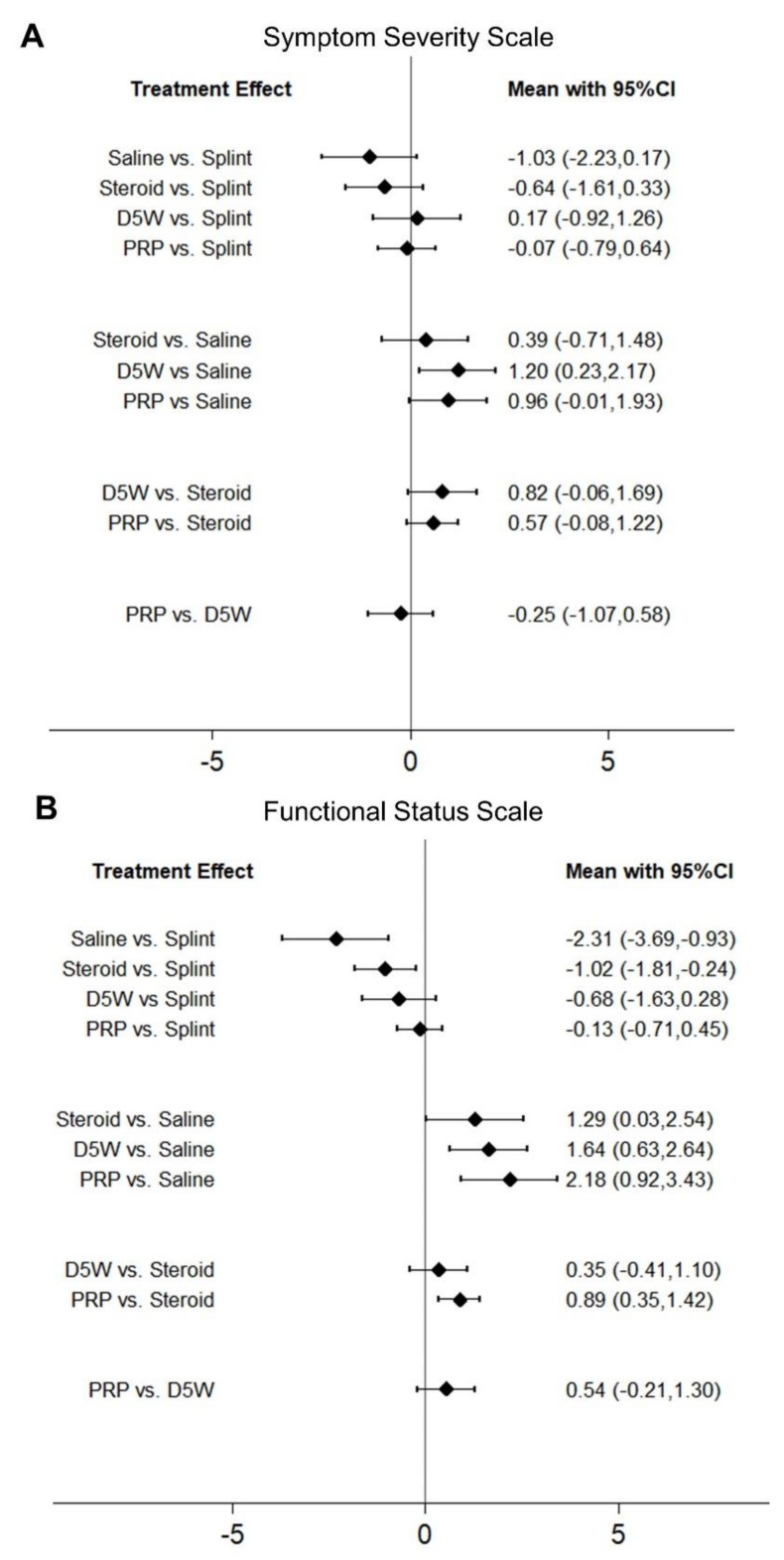
Forest plot of the network comparison of the standardized mean difference between different subgroups in terms of the (**A**) symptom severity scale and (**B**) functional status scale of the Boston Carpal Tunnel Questionnaire. *PRP, platelet-rich plasma; D5W, 5% dextrose.*

A forest plot of pairwise meta-analysis for the SMDs between the different therapeutic regimens is presented in Figure 2. For the network meta-analysis, the network graph is shown in Figure S3, and its forest plot is demonstrated in Figure 3. The league tables for pairwise and network meta-analyses of SSS and functional status scale (FSS) are summarized in Tables S1 and S2, respectively.

In the pairwise meta-analysis (Figure 2), PRP injection was superior to corticosteroid and saline injections, with SMDs of 0.71 (95% confidence interval [CI], 0.62 to 0.80; I^2^ = 93.0%) and 1.05 (95% CI, 0.92 to 1.18), respectively. The difference of SMD between PRP injection and splinting was 0.01, with no significance (95% CI, −0.10 to 0.11; I^2^ = 98.8%). D5W was better than corticosteroid and saline injections, with SMDs of 0.67 (95% CI, 0.51 to 0.82) and 1.11 (95% CI, 0.94 to 1.29), respectively. There was a significant difference of PRP injection vs. D5W injection, with a SMD of −0.48 (95% CI, −0.63 to −0.34).

In the network meta-analysis, the SMDs comparing PRP injection with splinting, saline injection, corticosteroid injection, and D5W injection were −0.07 (95% CI, −0.79 to 0.64), 0.96 (95% CI, −0.01 to 1.93), 0.57 (95% CI, −0.08 to 1.22), and −0.25 (95% CI, −1.07 to 0.58), respectively (Figure 3). Furthermore, the SMDs comparing D5W injection with splinting, saline injection, and corticosteroid injection were 0.17 (95% CI, −0.92 to 1.26), 1.20 (95% CI, 0.23 to 2.17), and 0.82 (95% CI, −0.06 to 1.69), respectively. Only the comparison of D5W injection vs. saline injection in this network reached statistical significance. The inconsistency analysis showed that all CIs for the difference between direct and indirect comparisons crossed the value of zero inconsistency, indicating that there is no significant inconsistency between direct and indirect comparisons.

**Figure 4:**
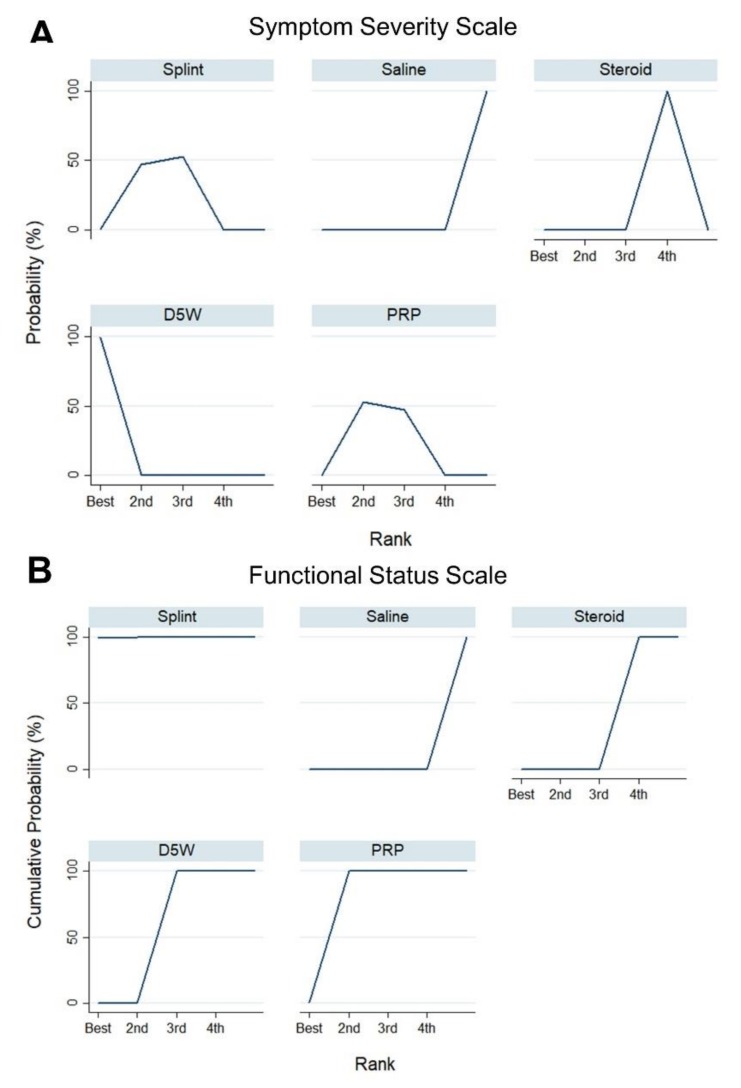
Ranking probabilities for different subgroups based on the standardized mean difference between different subgroups in terms of the (**A**) symptom severity scale and (**B**) functional status scale of the Boston Carpal Tunnel Questionnaire. *PRP, platelet-rich plasma; D5W, 5% dextrose.*

Ranking of the treatment effect for SSS is presented in Figure 4. The results of the simulation of rank probabilities showed that D5W injection had the highest probability (99.8%) to be the best treatment, whereas PRP injection (52.8%) had the largest chance to be the second best treatment. Likewise, splinting, corticosteroid injection, and saline injection were likely to be the third best (with a probability of 52.8%), fourth best (with a probability of 100%), and the least effective (with a probability of 100%) treatments, respectively.

### 2.4. Comparison of SMDs of FSS among Different Treatments

In the pairwise meta-analysis (Figure 2), PRP injection was better than D5W (SMD, 0.59; 95% CI, 0.41 to 0.77) and corticosteroid (SMD, 0.92; 95% CI, 0.83 to 1.01; I^2^ = 95.0%) injections but was less effective than splinting (SMD, −0.15; 95% CI, −0.25 to −0.04; I^2^ = 98.0%). D5W injection was superior to saline (SMD, 1.64; 95% CI, 1.46 to 1.82) and corticosteroid (SMD, 0.39; 95% CI, 0.25 to 0.53) injections.

In the network meta-analysis, the SMDs comparing PRP injection with splinting, corticosteroid injection, and D5W injection were −0.13 (95% CI, −0.71 to 0.45), 0.89 (0.35 to 1.42), and 0.54 (−0.21 to 1.30), respectively. Moreover, the SMDs comparing D5W injection with splinting and corticosteroid injection were −0.68 (95% CI, −1.63 to 0.28) and 0.35 (95% CI, −0.41 to 1.10), respectively (Figure 3). PRP and D5W injections were superior to saline injection, with SMDs of 2.18 (95% CI, 0.92 to 3.43) and 1.64 (95% CI, 0.63 to 2.64), respectively. Similar to the analyses of SMDs of SSS, no significant inconsistency was observed between direct and indirect comparisons for the SMDs of FSS.

The ranking of the treatment effect according to FSS is presented in Figure 4. The results of the simulation of rank probabilities showed that splinting had the highest probability (99.5%) to be the best treatment. PRP, D5W, corticosteroid, and saline injections were likely to be the second best (with a probability of 99.5%), third best (with a probability of 100.0%), fourth best (with a probability of 100.0%), and least effective (with a probability of 100.0%) treatments, respectively.

### 2.5. Publication Bias

**Figure 5:**
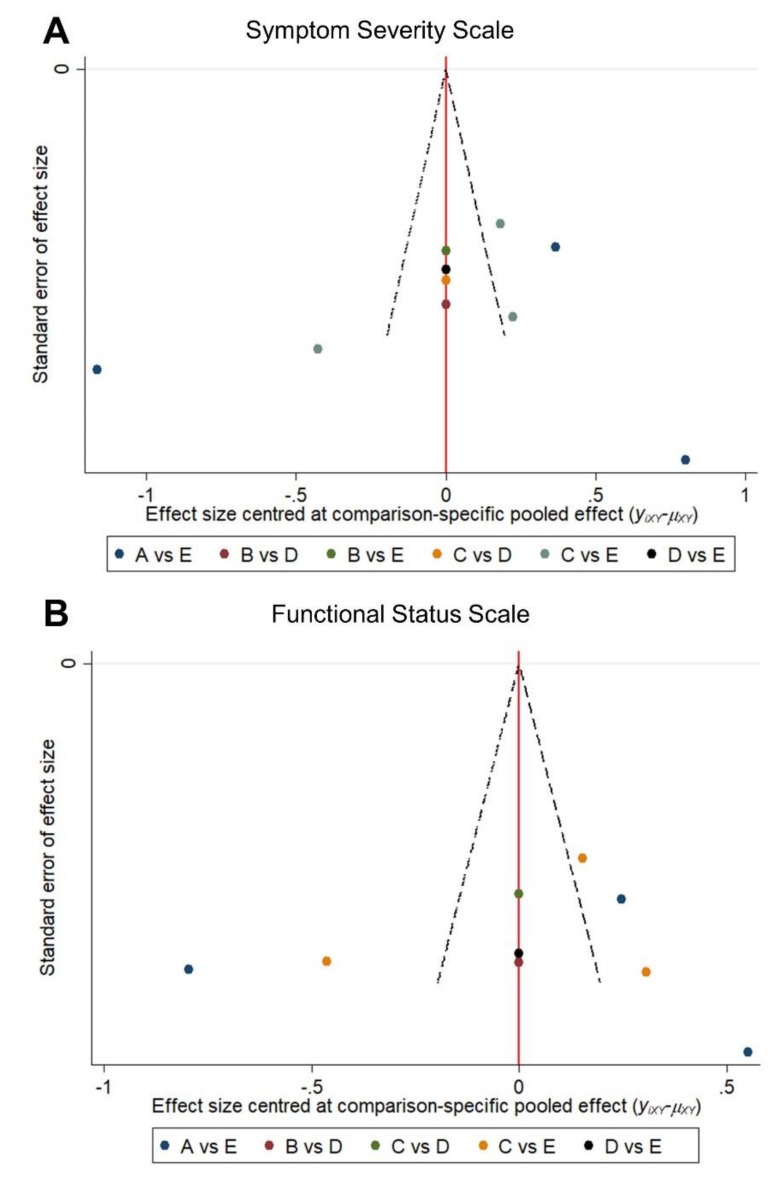
Funnel plot for the comparisons of the standardized mean difference between different subgroups in terms of the (**A**) symptom severity scale and (**B**) functional status scale of the Boston Carpal Tunnel Questionnaire. A: splinting; B: saline; C: steroid; D: 5% dextrose; E: platelet-rich plasma.

The funnel plots for SSS and FSS (Figure 5) revealed a symmetric distribution of the inter-group comparisons of the SMDs. The Egger’s test did not reveal a significantly small study bias (*p* = 0.386 for SSS and 0.692 for FSS, respectively).

## 3. Discussion

This meta-analysis incorporated direct and indirect evidence from relevant clinical trials, yielding several important findings on regenerative injections in CTS. First, considering the effectiveness of symptom relief, D5W injection was likely to be the best alternative, followed by PRP injection. Second, splinting ranked higher than PRP and D5W injections in terms of functional recovery. Third, corticosteroid and saline injections ranked fourth and fifth, respectively, with respect to clinical effectiveness in providing symptom and function improvement.

Several meta-analyses have investigated the effects of injection therapies for CTS. Most of them focused on comparisons of various local injection techniques instead of on different regimens. In 2007, a systematic review [14] including 671 participants from 12 studies concluded that corticosteroid injection provided short-term symptom relief in contrast to placebo injection. In 2015, a network meta-analysis [35] with 633 patients from 10 studies compared the effectiveness of corticosteroid injections, using different injection approaches. Their analysis indicated that the ultrasound-guided in-plane injection method through the ulnar aspect of the wrist was the best approach in CTS treatment. In 2018, another meta-analysis [36] including 181 hands from three randomized trials attempted to determine whether ultrasound guidance provided better clinical outcomes. The authors reported that ultrasound-guided corticosteroid injection was superior to the landmark-guided approach for the improvement of symptom severity but not functional status and electrophysiological findings. While regenerative medicine is an emerging field in the treatment of musculoskeletal diseases, only one systematic review [37] proposed PRP injection to be a potential alternative treatment for mild to moderate CTS. Nevertheless, there is lack of quantitative analysis incorporating evidence from multiple studies to demonstrate the effectiveness of regenerative injection for CTS.

In our network meta-analysis, the simulation of therapeutic effectiveness in terms of symptom relief revealed D5W and PRP injections to be the best and second best treatment, respectively. Although little statistical significance was observed in the network comparisons of SMDs between regenerative injections and other treatments, the trends of effect sizes were in accordance with those in the pairwise comparisons (Table S1). Low-concentration dextrose had been first studied as a solution for the hydro-location of the needle tip during regional anesthesia, revealing no decrease of block effect with additional use of D5W [38]. Later, several case reports were published on the potential utility of D5W injection in entrapment neuropathies [39,40]. Although there is insufficient evidence from animal and histopathological studies for the exact beneficial mechanism of D5W, a commonly accepted pathway is through the inhibition of transient receptor potential vanilloid receptor-1 (TRPV1) [41]. The TRPV1 receptor can trigger nociceptive sensation by evoking an action potential from the peripheral nerve endings, and is susceptible to inflammatory stimuli [41]. Our results might also reflect the crucial influence of neurogenic inflammation on CTS symptoms.

On the basis of our review, the evidence on PRP injection for CTS was more abundant than that on D5W injection. PRP, which contains high concentrations of autologous growth factors, is commonly used to accelerate tissue repair in various kinds of musculoskeletal disorders [42,43]. Several in vitro and animal studies have revealed the potential of PRP in remyelination, axonal regeneration, and angiogenesis of injured peripheral nerves [44]. Our quantitative analysis further demonstrated a favorable outcome of PRP for symptom relief in mild to moderate CTS. The main concern about PRP application in CTS treatments lies on how it is prepared and which portion of plasma has been extracted for injection. Unlike D5W, which contains a fixed concentration of dextrose, the final platelet concentration in PRP varies across patients and different preparation systems. Further, because increased expression of inflammatory cytokines (e.g., transforming growth factor-β) is commonly identified in endothelial cells and subsynovial connective tissues of the median nerves in patients with CTS [45], injection of leukocyte-rich PRP may potentiate neurogenic inflammation and worsen the symptoms. However, among our retrieved trials, the obtained information was not detailed enough to resolve the aforementioned concern.

There was one finding worth mentioning concerning the comparative effectiveness of symptom relief between D5W and PRP injections. The volume of D5W (3–5 mL) was higher than that of PRP (1–3 mL) in our included studies. Additionally, ultrasound guidance was used in the two trials of D5W, but not in all studies using PRP. A cadaveric study has demonstrated that a bolus saline injection could effectively reduce the peak gliding resistance of the median nerve [46]. Another randomized controlled trial pointed out that precise hydro-dissection of the median nerve using saline under ultrasound guidance yielded better clinical outcomes than subcutaneous saline injection [47]. Therefore, the observed superiority of D5W over PRP (with respect to CTS symptoms) in this meta-analysis may be partly derived from a higher injection volume and the mechanical effect of nerve hydro-dissection guided by ultrasound imaging.

Concerning the functional status of patients with CTS, our analysis revealed that splinting is the best treatment, followed by PRP injection. The mechanism of the effect of splinting lies in reducing the intra-tunnel pressure through the neutral positioning of the wrist. Although splinting is not likely to provide short-term (e.g., one month) benefits compared with no treatment, its long-term effect has rarely been studied [8]. Regenerative injections may cause a rapid decrease of symptom severity; however, functional improvement still depends on the recovery of muscle endurance and strength, which requires adequate rest and proper wrist positioning, with the use of a splint. Further, although PRP injection ranked second in effectiveness in terms of functional improvement, the patients in the two included PRP studies [23,29] had additional splinting after the injection. An earlier randomized controlled trial has revealed that splinting combined with corticosteroid injection was superior to corticosteroid injection alone, in terms of functional recovery and symptom relief in CTS [48]. Therefore, it is reasonable to speculate that post-injection splinting contributed to the functional outcome in the PRP group.

The present meta-analysis has several clinical implications. First, D5W and PRP injections can be considered useful regimens for the treatment of CTS of mild to moderate severity because both methods yield better effectiveness in terms of symptom relief and functional improvement than corticosteroid and saline injections. Second, the mechanical effect of hydro-dissection may substantially contribute to the benefits derived from D5W injection. Hence, injection of D5W with an enough volume (e.g., 5 mL) and under ultrasound guidance is recommended. Third, splinting provides proper positioning and adequate rest of the wrist in patients with CTS, and seems crucial for functional recovery. Post-injection splinting should be considered in combination with regenerative injections.

This meta-analysis has several limitations. First, because not every included study documented electrophysiological findings and ultrasonographic nerve cross-sectional area measurements, we were not able to compare the changes of those parameters after treatments. Second, the timing of outcome assessment varied, and the third month (or close to it) after injection was the most suitable point because of the availability of data for this time point in almost all included trials. However, the long-term (> 6 months) effectiveness of regenerative injection could not be analyzed in the present meta-analysis. Third, some of the enrolled studies, especially those investigating D5W injection, were from the same research group. The generalization of results might be limited by some systemic factors, such as ethnicity and disease severity. Therefore, we strongly believe that more studies are needed to examine whether similar efficacy can be observed in various patient populations. Fourth, all the patients enrolled in the present meta-analysis only had CTS of mild to moderate severity. Considering the regenerative potential of both proposed regimens (especially for PRP), it would be of great interest to apply them on severe cases or those with an unsatisfactory outcome after CTR in future studies. 

## 4. Conclusions

D5W and PRP injections are more effective than splinting, corticosteroid injection, or saline injection for relieving the symptoms of CTS. D5W and PRP injections might not be superior to splinting in terms of functional recovery. More studies are needed in the future, to explore the long-term effectiveness of regenerative injections in the treatment of CTS.

## 5. Methods

### 5.1. Literature Search

This review was conducted in accordance with the PRISMA (Preferred Reporting Items for Systematic Reviews and Meta-Analyses) guidelines [49]. PubMed and Embase were used as the two main electronic databases for the literature search, which covered records from the earliest time to July 2019, without language limitation. The references of relevant articles and the registry of the clinical trials, such as ClinicalTrials.gov, were also manually searched for eligible studies. The following keywords were used in different combinations: “carpal tunnel syndrome”, “platelet-rich plasma”, and “dextrose”. Two authors independently read the titles and abstracts of the retrieved literature and reviewed the full text of articles, potentially fulfilling our inclusion criteria. The search strategy is provided in Appendix A.

### 5.2. Inclusion and Exclusion Criteria

The inclusion criterion was being a clinical study comparing regenerative injections (D5W and PRP) with non-surgical treatments for CTS. The exclusion criteria were (1) being case reports/series, reviews, and non-human trials; (2) lacking quantitative outcome assessment; (3) lacking a control group; and (4) being studies on other peripheral nerve entrapment syndromes (other than CTS).

### 5.3. Data Extraction and Quality Assessment

Two authors independently evaluated the quality of the included trials by using the Cochrane risk-of-bias tool for randomized controlled trials [50]. Six aspects were evaluated, including (1) random sequence generation, (2) allocation concealment, (3) blinding of participants and personnel, (4) incomplete outcome data, (5) selective reporting, and (6) other biases. Each item is appraised as having a low, high, or unclear risk of bias. Any discrepancy of opinions between the two authors was resolved by discussion or through the decision of the corresponding author.

### 5.4. Data Synthesis and Analysis

The following data were retrieved from the included studies: first author, publication year, study design, sample size, disease severity, follow-up timing and duration, regenerative injection regimen, guiding technique, and details of the comparative treatments. The main outcome was the SMD of the scores in the BCTS before and after treatments [51]. The SSS and FSS were analyzed separately. If the study only used the VAS for pain for symptom assessment, we considered the VAS as a surrogate for the SSS. Because the follow-up timing and duration were different among the studies, we chose the assessments at baseline and at or close to the 3rd month post-injection, for analysis.

In the pairwise analysis, the random-effect model was chosen because the regenerative injection regimens and their counterpart treatments varied across the trials. The heterogeneity across the studies was expressed by using the I2 statistics and analyzed by using the Cochrane Q test [52]. A value of I2 > 50% was considered statistically significant heterogeneity [52]. For the network meta-analysis, a generalized linear mixed model was used to combine the direct and indirect comparisons. The loop inconsistency model was used to examine whether inconsistency existed between direct and indirect evidence [53]. Ranking probabilities of the SMD of each treatment were acquired by using simulation and presented by using ranking probability curves [53]. The funnel plot method and Egger’s test were used to investigate the potential existence of publication bias [53], indicating that studies with larger sample sizes and significant results are more likely to be published than those with small participant numbers and insignificant outcomes. The statistical software package Stata (StataCorp. 2015. Stata Statistical Software: Release 14. StataCorp LP, College Station, TX, USA) was employed for all analyses, and *p* < 0.05 was considered statistically significant.

## Figures and Tables

**Table 1:** Summary: the characteristics of included studies using regenerative injection (comprising 5% dextrose and platelet-rich plasma) for treating carpal tunnel syndrome.

Author, Year	Study Design	Inclusion Criteria	Treatment Allocation	Participant Characteristics			Symptom Duration (Months)	Disease Severity	Randomization	Blinding	Outcome Measure	Follow-Up (Week)
				Number of Participants (Wrists)	Mean Age (Year)	Female (%)						
Uzun et al. 2017	Prospective quasi-experimental	Clinical + EDS	PRP	20 (20)	48.8 ± 5.8	80	NA	Minimal to mild	No	No	BCTQ, EDS	12,24
			Triamcinolone	20 (20)	48.5 ± 6.1	80						
Wu et al. 2017(Mayo Clin Proc)	RCT	Clinical + EDS	D5W	25 (30)	58.4 ± 2.3 *	86.7	44.5 ± 7.5 *	Mild to moderate	Computer-generated randomization	Blinded participants and outcome assessors	VAS, BCTQ, EDS, CSA of MN, Global assessment of treatment results	4,12,24
			Normal saline	24 (30)	58.1 ± 1.9 *	80	44.4 ± 5.5 *					
Wu et al. 2017(Sci Rep)	RCT	Clinical + EDS	PRP	30 (30)	57.87 ± 1.5 *	90	34.43 ± 5.6 *	Mild to moderate	Computer-generated randomization	Blinded outcome assessors	VAS, BCTQ, EDS, CSA of MN, Finger pinch strength	4,12,24
			Splinting	30 (30)	54.27 ± 1.3 *	83.3	30.70 ± 6.0 *					
Malahias et al. 2018	RCT	Clinical	PRP	26 (26)	60.4 ± 14.3	NA	NA	Mild to moderate	Closed envelope method	Blinded outcome assessors	VAS, Q-DASH, Delta- CSA of MN	4,12
			Normal saline	24 (24)	57.1 ± 16.1							
Raeissadat et al. 2018	RCT	Clinical + EDS	PRP + splinting	21 (21)	51.2 ± 9.8	100	13.7 ± 11.5	Mild to moderate	Online randomization website	No	VAS, BCTQ, EDS	10
			Splinting	20 (20)	47.2 ± 7.1	100	14.1 ± 8.5					
Wu et al. 2018	RCT	Clinical + EDS	D5W	27 (27)	58.6 ± 2.2 *	81.4	46.8 ± 8.9 *	Mild to moderate	Computer-generated randomization	Blinded outcome assessors	VAS, BCTQ, EDS, CSA of MN, global assessment of treatment results	4,12,16,24
			Triamcinolone	27 (27)	54.3 ± 2.0 *	77.7	45.6 ± 9.4 *					
Güven et al. 2019	Prospective quasi-experimental	Clinical + EDS	PRP + Splinting	18 (20)	47.5	94.4	72	Mild to moderate	No	No	BCTQ, EDS, CSA of MN, monofilament testing score, static 2PD testing score, dynamic 2PD testing score	4
			Splinting	12 (20)	50.0	91.6	60					
Atwa et al. 2019	Prospective quasi-experimental	Clinical + EDS	PRP	18 (18)	38.5 ± 8.0	88.8	14 ± 9	Mild to moderate	No	No	VAS, BCTQ, EDS	4,12
			Methylprednisolone	18 (18)	36.6 ± 8.8	88.8	19 ± 11					
Shen et al. 2019	RCT	Clinical + EDS	PRP	26 (26)	56.8 ± 1.7 *	96.2	58.3 ± 16.2 *	Moderate	Computer-generated randomization	Blinded outcome assessors	BCTQ, EDS, CSA of MN	4,12,24
			D5W	26 (26)	58.5 ± 2.1 *	84.6	37.5 ± 8.0 *					
Senna et al. 2019	RCT	Clinical + EDS	PRP	43 (43)	38.3 ± 6.4	81.4	NA	Mild to moderate	Block randomization	Blinded participants and outcome assessors	VAS, BCTQ, EDS, CSA of MN, Paresthesia, Phalen’s maneuver, and Tinel’s sign	4,12
			Methylprednisolone	42 (42)	40.7 ± 9.4	85.7						

BCTQ: Boston carpal tunnel syndrome questionnaire, VAS: visual analogue scale, PRP: platelet-rich plasma, D5W: 5% dextrose; NA: not available, RCT: randomized controlled trial, EDS: electrodiagnostic study, CSA: cross-sectional area, MN: median nerve, Q-DASH: quick disabilities of the arm, shoulder, and hand scale, 2PD: two-point discrimination, Delta-CSA: cross sectional area difference of the median nerve’s surface at the tunnel’s inlet, minus the median nerve’s surface proximal to the tunnel and over pronator quadratus. NOTE: data are presented as *n* or mean ± standard deviation; * presented as mean ± standard error.

**Table 2:** Summary: the intervention details of included studies using regenerative injection (comprising 5% dextrose and platelet rich plasma) for treating carpal tunnel syndrome.

Author, Year	Intervention	Intervention Regiment	Guidance Method	Injection Site	Adverse Effect
Uzun et al. 2017	PRP vs. Corticosteroid	PRP:2 mL PRP * Corticosteroid:40 mg/1 ml triamcinolone	Palpation-guided	1 cm proximal to the distal wrist-flexion crease	No complication
Wu et al. 2017(Mayo Clin Proc)	Prolotherapy vs. Placebo	Prolotherapy:5 mL of 5% dextrose Placebo:5 mL normal saline	US-guided	Carpal tunnel inlet	No complication
Wu et al. 2017(Sci Rep)	PRP vs. Splinting	PRP:3 mL RegenKit-THT-1 Splinting:overnight ≥ 8 h daily	US-guided	Carpal tunnel inlet	No complication
Malahias et al. 2018	PRP vs. Placebo	PRP:2 mL PRP * Placebo:2 mL 0.9% normal saline	US-guided	Carpal tunnel inlet	No complication
Raeissadat et al. 2018	PRP vs. Splinting	PRP:0.8–1 mL Rooyagen kit, 0.5 mL lidocaine, night splint Splinting:overnight for 8 weeks	Palpation-guided	Distal carpal skin crease	4 with pruritus, 1 with post-injection pain and 1 with burning sensation
Wu et al. 2018	Prolotherapy vs. Corticosteroid	Prolotherapy:5 mL of 5% dextrose Corticosteroid:3 ml of triamcinolone (10 mg/mL), 2 mL normal saline	US-guided	Carpal tunnel inlet	No complication
Güven et al. 2019	PRP vs. Splinting	PRP:1 mL PRP*, night splinting, activity modification for 4 weeks Splint:night splinting, activity modification for 4 weeks	US-guided	Not specified	No complication
Atwa et al. 2019	PRP vs. Corticosteroid	PRP:2 mL PRP * Corticosteroid:40 mg/1 ml methylprednisolone	Palpation-guided	1 cm proximal to the distal wrist-flexion crease	Not mentioned
Shen et al. 2019	PRP vs. Prolotherapy	PRP:3 mL RegenKit-THT-1 Prolotherapy:3 mL of 5% dextrose	US-guided	Carpal tunnel inlet	No severe complication
Senna et al. 2019	PRP vs. Corticosteroid	PRP:2 mL GD medical pharma Corticosteroid:40 mg/1 ml methylprednisolone	US-guided	Carpal tunnel inlet	No complication

PRP: platelet-rich plasma, US-guided: ultrasound-guided. * Brand of PRP kit was not mentioned.

## Supplementary Materials

The following are available online at https://www.mdpi.com/1424-8247/13/3/49/s1, **Figure S1.** Summary of the quality assessment of the included studies: green indicates low risk of bias; red, high risk of bias; and yellow, unclear risk of bias. **Figure S2.** Quality assessment graph: green indicates low risk of bias; red, high risk of bias; and yellow, unclear risk of bias. **Figure S3.** Network graphs for the comparison of the standardized mean difference between different subgroups in terms of the (A) symptom severity scale and (B) functional status scale of the Boston Carpal Tunnel Questionnaire. *PRP, platelet-rich plasma; D5W, 5% dextrose.* **Table S1.** League table for the results of the meta-analysis according to the symptom severity scale of the Boston Carpal Tunnel Questionnaire. **Table S2.** League table for the results of the meta-analysis according to the functional status scale of the Boston Carpal Tunnel Questionnaire.

## Appendix A: Search Strategy

PubMed

1. (carpal tunnel syndrome[Title/Abstract]) AND dextrose[Title/Abstract]
2. (carpal tunnel syndrome[Title/Abstract]) AND platelet rich plasma[Title/Abstract]
3. 1 OR 2

Embase

1. (carpal tunnel syndrome[Title/Abstract]) AND dextrose[Title/Abstract]
2. (carpal tunnel syndrome[Title/Abstract]) AND platelet rich plasma[Title/Abstract]
3. 1 OR 2
