# Supplementary material for: Regenerative Injections Including 5% Dextrose and Platelet-Rich Plasma for the Treatment of Carpal Tunnel Syndrome: A Systematic Review and Network Meta-Analysis
Source: Pharmaceuticals (Basel). 2020 Mar 18;13(3):49. doi: 10.3390/ph13030049 (PMC7151714; doi:10.3390/ph13030049)
Supplement: Supplementary file 1 [file pharmaceuticals-13-00049-s001.zip › supplementary.v7/pharmaceuticals-725173-supplementary.docx]

Regenerative Injections Including 5% Dextrose and Platelet-rich Plasma for the Treatment of Carpal Tunnel Syndrome: A Systematic Review and Network Meta-analysis

Supplementary material:


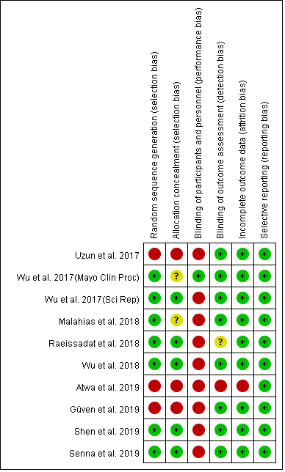


**Figure S1.** Summary of the quality assessment of the included studies: green indicates low risk of bias; red, high risk of bias; and yellow, unclear risk of bias.


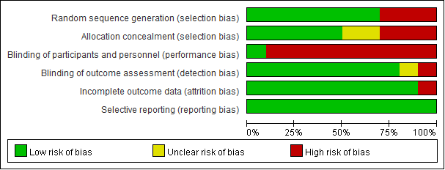


**Figure S2.** Quality assessment graph: green indicates low risk of bias; red, high risk of bias; and yellow, unclear risk of bias.


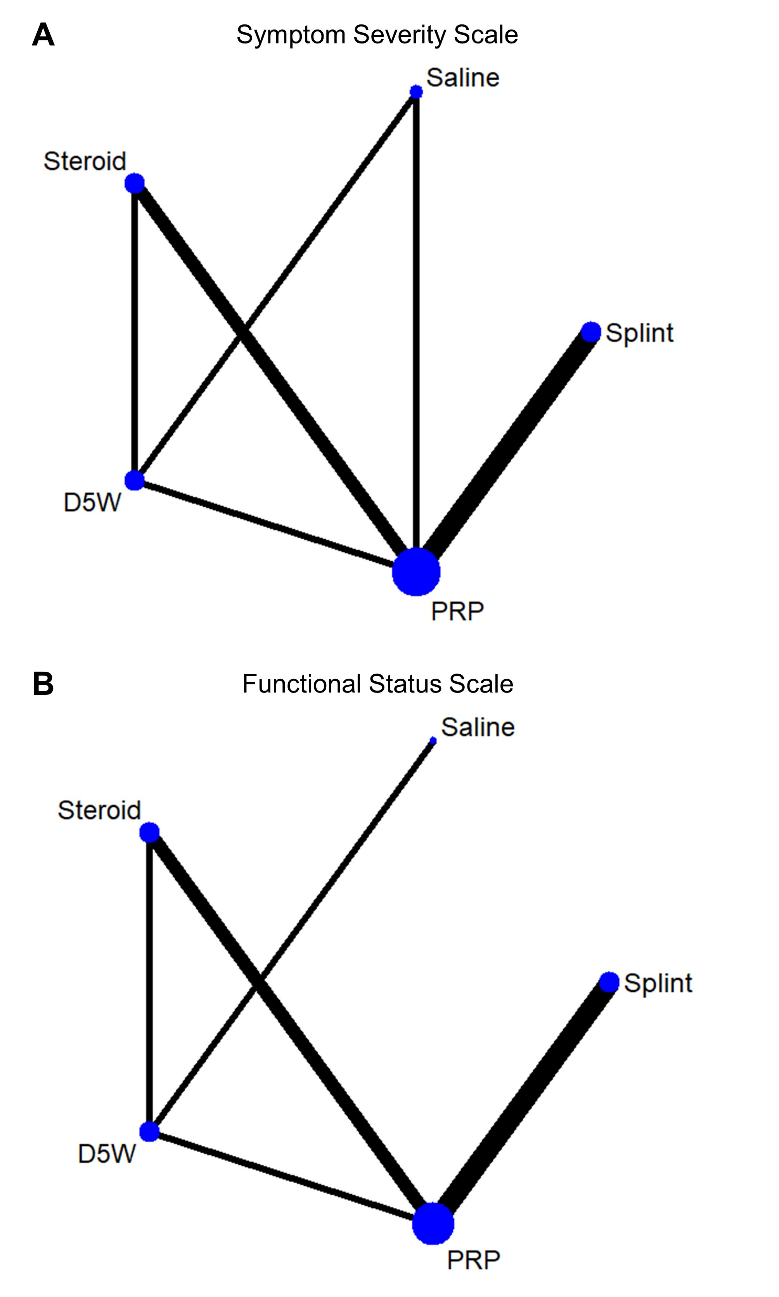


**Figure S3.** Network graphs for the comparison of the standardized mean difference between different subgroups in terms of the (A) symptom severity scale and (B) functional status scale of the Boston Carpal Tunnel Questionnaire. *PRP, platelet-rich plasma; D5W, 5% dextrose.*

**Table S1.** League table for the results of the meta-analysis according to the symptom severity scale of the Boston Carpal Tunnel Questionnaire.

| **PRP** | **-0.48(-0.63,-0.34)** | **1.05(0.92,1.18)** | **0.71(0.62,0.80)** | **0.01(-0.10,0.11)** |
| --- | --- | --- | --- | --- |
| -0.25(-1.07,0.58) | **D5W** | 1.11(0.94,1.29) | 0.67(0.51,0.82) | NA |
| 0.96(-0.01,1.93) | 1.20(0.23,2.17) | **Saline** | NA | NA |
| 0.57(-0.08,1.22) | 0.82(-0.06,1.69) | 0.39(-0.71,1.48) | **Steroid** | NA |
| -0.07(-0.79,0.64) | 0.17(-0.92,1.26) | -1.03(-2.23,0.17) | -0.64(-1.61,0.33) | **Splint** |

All data are presented as pooled standardized mean differences of symptom severity scale with 95% CIs. The left lower part is the findings of the network meta-analysis; the right upper part is the finding of the pairwise meta-analyses. *PRP, Platelet rich plasma; D5W, 5% dextrose; NA, not available.*

**Table S2.** League table for the results of the meta-analysis according to the functional status scale of the Boston Carpal Tunnel Questionnaire.

| **PRP** | **0.59(0.41,0.77)** | **NA** | **0.92(0.83,1.01)** | **-0.15(-0.25,-0.04)** |
| --- | --- | --- | --- | --- |
| 0.54(-0.21,1.30) | **D5W** | 1.64(1.46,1.82) | 0.39(0.25,0.53) | NA |
| 2.18(0.92,3.43) | 1.64(0.63,2.64) | **Saline** | NA | NA |
| 0.89(0.35,1.42) | 0.35(-0.41,1.10) | 1.29(0.03,2.54) | **Steroid** | **NA** |
| -0.13(-0.71,0.45) | -0.68(-1.63,0.28) | -2.31(-3.69,-0.93) | -1.02(-1.81,-0.24) | **Splint** |

All data are presented as pooled standardized mean differences of functional status scale with 95% CIs. The left lower part is the findings of the network meta-analysis; the right upper part is the finding of the pairwise meta-analyses. *PRP, Platelet rich plasma; D5W, 5% dextrose; NA, not available.*
