# Supplementary figures and images for: Regenerative Injections Including 5% Dextrose and Platelet-Rich Plasma for the Treatment of Carpal Tunnel Syndrome: A Systematic Review and Network Meta-Analysis
Source: Pharmaceuticals (Basel). 2020 Mar 18;13(3):49. doi: 10.3390/ph13030049 (PMC7151714; doi:10.3390/ph13030049)

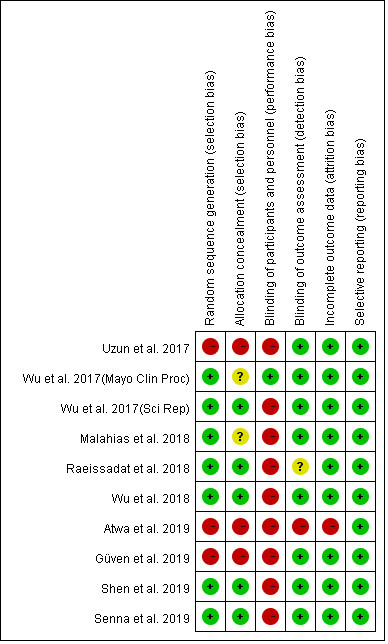

Supplement: Supplementary file 1 [file pharmaceuticals-13-00049-s001.zip › supplementary.v7/Figure S1.jpg]

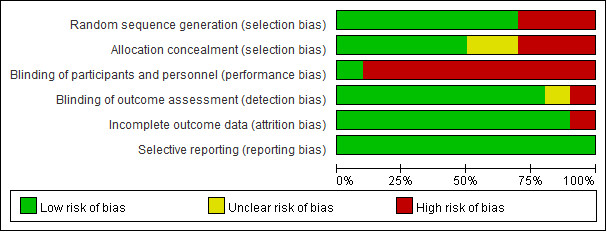

Supplement: Supplementary file 1 [file pharmaceuticals-13-00049-s001.zip › supplementary.v7/Figure S2.jpg]

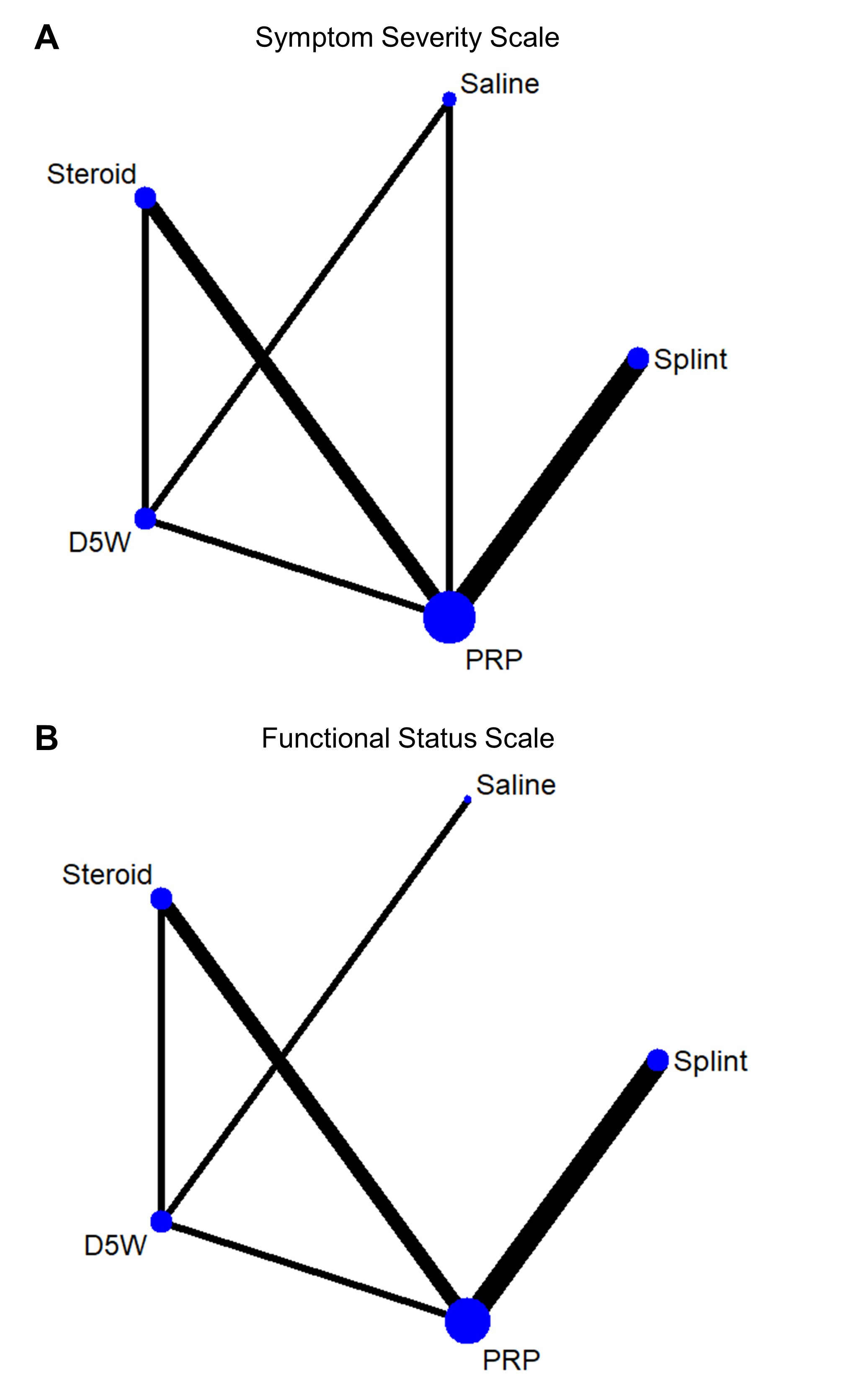

Supplement: Supplementary file 1 [file pharmaceuticals-13-00049-s001.zip › supplementary.v7/Figure S3.jpg]
